# Untargeted metabolomics profiling of oat (*Avena sativa* L.) and wheat (*Triticum aestivum* L.) infested with wheat stem sawfly (*Cephus cinctus* Norton) reveals differences associated with plant defense and insect nutrition

**DOI:** 10.3389/fpls.2024.1327390

**Published:** 2024-01-24

**Authors:** Megan S. Hager, Megan L. Hofland, Andrea C. Varella, Brian Bothner, Hikmet Budak, David K. Weaver

**Affiliations:** ^1^ Department of Plant Sciences and Plant Pathology, Montana State University, Bozeman, MT, United States; ^2^ Wheat Stem Sawfly Laboratory, Department of Land Resources and Environmental Sciences, Montana State University, Bozeman, MT, United States; ^3^ Corteva Agriscience™, Woodstock Research and Development Centre, Tavistock, ON, Canada; ^4^ Department of Chemistry and Biochemistry, Montana State University, Bozeman, MT, United States; ^5^ Department of Agriculture, Arizona Western College, Yuma, AZ, United States

**Keywords:** metabolomics, wheat stem sawfly, host plant resistance, insect feeding, *Triticum aestivum*, *Avena sativa*

## Abstract

**Introduction:**

Wheat stem sawfly (WSS), Cephus cinctus Norton, is a major pest of common bread wheat (Triticum aestivum L.) and other cultivated cereals in North America. Planting of cultivars with solid stems has been the primary management strategy to prevent yield loss due to WSS infestation, however expression of this phenotype can vary depending on environmental conditions and solid stems hinder biological control of WSS via braconid parasitoids Bracon cephi (Gahan) and Bracon lissogaster Muesebeck. In the hollow stems of oat (Avena sativa L.), WSS larvae experience 100% mortality before they reach late instars, but the mechanisms for this observed resistance have not been characterized.

**Objective:**

The objective of this study was to explore additional sources of resistance outside of the historic solid stem phenotype.

**Methods:**

Here, we use an untargeted metabolomics approach to examine the response of the metabolome of two cultivars of oat and four cultivars of spring wheat to infestation by WSS. Using liquid chromatography-mass spectrometry (LC-MS), differentially expressed metabolites were identified between oat and wheat which were associated with the phenylpropanoid pathway, phospholipid biosynthesis and signaling, the salicylic acid signaling pathway, indole-3-acetic acid (IAA) degradation, and biosynthesis of 1,4-benzoxazin-3-ones (Bxs). Several phospho- and galacto- lipids were found in higher abundance in oat, and with the exception of early stem solidness cultivar Conan, both species experienced a decrease in abundance once infested. In all wheat cultivars except Conan, an increase in abundance was observed for Bxs HMDBOA-glc and DIBOA-β-D-glucoside after infestation, indicating that this pathway is involved in wheat response to infestation in both solid and hollow stemmed cultivars. Differences between species in compounds involved in IAA biosynthesis, degradation and inactivation suggest that wheat may respond to infestation by inactivating IAA or altering the IAA pool in stem tissue.

**Conclusion:**

We propose that the species differences found here likely affect the survival of WSS larvae and may also be associated with differences in stem architecture at the molecular level. Our findings suggest pathways to focus on for future studies in elucidating plant response to WSS infestation.

## 1 Introduction

The wheat stem sawfly (WSS), Hymenoptera *Cephus cinctus* Norton, is a major pest of grasses in the Great Plains of North America. Adult female WSS use their saw-like ovipositor to lay their eggs in the lumen of stems and once hatched, larvae begin consuming parenchyma tissue in the stem lining. As the larvae grows it breaches nodes, damaging vascular tissues, impacting photosynthetic ability and leading to decreased head weight (Macedo et al., 2005; Delaney et al., 2010). The stem girdling action of mature larvae to prepare overwintering chambers also weakens standing stems, which makes them prone to lodging and thus makes recovery of heads difficult during harvest (Ainslie, 1920; Morrill et al., 1998; Sherman et al., 2010). The stem-boring activity of the WSS larval stage causes devastating yield loss in cultivated grass hosts such as spring and winter wheat (*Triticum aestivum* L.). However, when eggs are laid in the stems of wild oat (*Avena fatua* L.) and cultivated oat (*Avena sativa* L.), larvae are unable to complete development (Criddle, 1917; Criddle, 1923; Farstad, 1940; Roemhild, 1954; Sing, 2002). Although cultivated oat exhibits total resistance to WSS, the mechanism behind this resistance remains unknown (Weaver et al., 2004).

In spring wheat, the solid stem phenotype is utilized by producers as the primary defense against WSS, but it does not prevent infestation or stem cutting completely and may decrease success of biological control efforts (Rand et al., 2012; Rand et al., 2020). Solid stems are filled with pith which appears to hinder oviposition, larval growth and maturation. Solid stems do not completely inhibit WSS and stem cutting as high as 30% has been reported for solid and semi-solid stemmed spring wheat cultivars (Sherman et al., 2010). While stem solidness is a relatively stable phenotype in some spring and winter wheat cultivars (Subedi et al., 2021), it is often negatively affected by light quality and sowing density (Roemhild, 1954; Nilsen et al., 2016; Beres et al., 2017; Nilsen et al., 2017). There is also evidence that braconid parasitoids *Bracon cephi* (Gahan) and *Bracon lissogaster* Muesebeck (Hymenoptera: Braconidae) cause less mortality to WSS larvae in solid stemmed cultivars compared to their hollow stemmed counterparts, indicating that continued use of solid stemmed cultivars may have a negative effect on populations of these parasitoids over time (Rand et al., 2012; Buteler et al., 2015; Rand et al., 2020). Host plant resistance against a specific pest can also promote secondary pest populations over time (Straub et al., 2020). It is important to support the tritrophic system of plant, WSS and parasitoid to increase success of control efforts, and it is therefore necessary to continue to explore sources of resistance in addition to the solid stem phenotype.

The stem solidness trait is highly heritable and is associated with the quantitative trait locus (QTL) *Qss.msub-3BL* (Cook et al., 2004). This QTL has been well studied, and several alleles conferring differing levels of stem solidness have been identified at this locus (Varella et al., 2017; Cook et al., 2019; Wong et al., 2022). The allele *Qss.msub-3BL.a* is associated with the hollow stemmed phenotype, while the allele *Qss.msub-3BL.b* confers stem solidness throughout plant development. A third allele, *Qss.msub-3BL.c* was identified in the cultivar ‘Conan’ (PI 607549), which has solid stems early in development with pith disappearing as the plant matures. WSS is known to target many grass species in addition to wheat including barley, oat and many native or introduced grasses, all of which have a hollow stemmed phenotype (Criddle, 1917; Wallace and McNeal, 1966; Cockrell et al., 2017; Achhami et al., 2020). Oat offers an opportunity to explore a source of complete resistance to WSS that appears unrelated to the stem solidness phenotype.

Female WSS will oviposit in wild oat as well as both small and large stemmed cultivated oat, but the larvae always die in the stem before reaching maturity (Criddle, 1917; Farstad, 1940; Holmes and Peterson, 1960; Holmes and Peterson, 1964; Wallace and McNeal, 1966; Sing, 2002; Weaver et al., 2004). This phenomenon has been discussed and documented for many years, and although research has explored the possibility of resistance due to physical differences between wheat and oat stems, the exact cause of larval mortality in oat remains unknown. In 1923, Criddle described “excessive sap” in oat stems as a possible cause of larval mortality, but this is likely due to environmental conditions as it was not replicable in dry environments (Criddle, 1923; Farstad, 1940). Though the outcome is likely not caused by free moisture, there is some evidence that larvae in oat stems do not begin development as readily as larvae in wheat. In oat, larvae are less likely to form extensive, continuous tunnels throughout the stem and they molt less frequently than larvae developing in wheat (Farstad, 1940). Past research on oat plant responses to WSS infestation and injury has focused on the possibility of resistance due to physical differences between wheat and oat stems (Roemhild, 1954). Tissue surrounding vascular bundles in the nodes of oat stems is composed of tough sclerenchyma tissue, as opposed to the more pliable parenchyma tissue found in wheat nodes. However, this structural difference is not enough to explain the resistance observed in oat, since some larvae are still able to penetrate multiple nodes as they move throughout the stem (Roemhild, 1954). These observations on larvae feeding on oat stem tissues seem to suggest that there may be nutritional deficiencies or perhaps more likely, compounds present in oat which have antifeedant or insecticidal activities. WSS larvae are adapted to feeding within a plant stem, which prevents the use of conventional methods of insect rearing to test the effects of specific compounds in an artificial diet. This has resulted in limited knowledge of the nutritional requirements of WSS larvae. While little is known about the identity of compounds in oat which are detrimental to WSS development, it has been determined that stems of oat and wheat do not differ in moisture or nitrogen content at earlier growth stages when WSS larvae are susceptible to mortality (McGinnis and Kasting, 1961). Additionally, both oat and wild oat are known to produce several active phenolic compounds in roots, shoots and seeds (Schumacher et al., 1983; Kato-Noguchi et al., 1994). In early development these are exuded allelopathic compounds, preventing establishment of seedlings of other species, and can also be induced in response to pest or pathogen attack at any growth stage (Pérez and Ormeño-Nuñez, 1991; Kato-Noguchi et al., 1994). Overall, little mechanistic research has been done to explore oat resistance to WSS and defining the potential molecular mechanisms of WSS resistance in oat represents an important step towards developing wheat cultivars with new forms of resistance to WSS.

In this study we use liquid-chromatography mass spectrometry (LC-MS) to evaluate the metabolites that define the physiological response of spring wheat and oat to WSS infestation. Montana spring wheat cultivars ‘Choteau’ (PI 633974), ‘Scholar’ (PI 607557), Conan and Reeder (PI 613586) were chosen for comparison against the oat cultivars Dane and Otana. A susceptible cultivar of oat has not yet been found, and both Dane and Otana are considered to be resistant. The cultivars Choteau and Scholar both have the allele *Qss.msub-3BL.b* associated with the solid stem phenotype, though Choteau exhibits greater expression of stem solidness and is considered more resistant to WSS than Scholar as it experiences lower rates of stem cuttting. Reeder is a susceptible hollow stemmed cultivar with the allele *Qss.msub-3BL.a* and Conan shows a unique phenotypic expression of stem solidness, being solid at early growth stages and losing pith as the plant matures. Resistance due to the Conan allele is also conferred through antixenosis, which causes WSS females to reduce the number of ovipositor insertions and lay fewer eggs (Sherman et al., 2010; Talbert et al., 2014; Varella et al., 2016). While we expect to find differences in metabolites between species, the goal of this research is to discover how these differences may be involved in the resistance of oat to WSS through a comparison of the small molecule profiles of infested oat and wheat plants with varying degrees of resistance.

## 2 Materials and methods

### 2.1 Experimental conditions and plant collection

Seeds from the two cultivars of oat and four cultivars of spring wheat (**Table 1**) were planted in 20.32 cm circular pots in a mix of MSU mix and Sunshine mix #1 in a 50:50 by volume ratio. MSU mix contained a composite of mineral soils from the Gallatin Valley, Canadian sphagnum peat moss and washed concrete sand in a 1:1:1 by volume ratio as in Varella et al. (2017). Sunshine Mix #1 consisted of a soil-less blend of Canadian Sphagnum peat moss and horticultural grade Perlite. For each cultivar six pots containing four seeds were planted for infestation and four pots also containing four seeds were planted for controls. Once germinated, plants were culled to three per pot and maintained under greenhouse conditions (22° ± 2° during the day and 20° ± 2° during the night, and photoperiod of 15L:9D h) at the Plant Growth Center at Montana State University. Plants were exposed to both natural and artificial light (GE Multivapor lamps; model MVR1000/C/U, GE Lighting, General Electric Co., Cleveland, Ohio). All plants were watered daily and fertilized once a week with Peters Professional® General Purpose Fertilizer (J.R. Peters, Inc., Allentown, Pennsylvania, United States) at 100 ppm in aqueous solution. Common greenhouse pests were controlled using ladybugs (*Hippodamia convergens* Guérin-Méneville) and mechanical removal by hand or by using a water spray to dislodge them. Plant damage due to greenhouse pests was negligible.

**Table 1:** Stem solidness and resistance of oat and wheat cultivars.

Species	Cultivar	Stem solidness
Oat (*A. sativa*)	Dane	Hollow, complete resistance
Oat (*A. sativa*)	Otana	Hollow, complete resistance
Spring wheat (*T. aestivum*)	Choteau	Solid, resistant
Spring wheat (*T. aestivum*)	Scholar	Semi-solid, semi-resistant
Spring wheat (*T. aestivum*)	Conan	Early solid, resistant
Spring wheat (*T. aestivum*)	Reeder	Hollow, susceptible

Stubble containing overwintering wheat stem sawfly larvae in diapause were collected in a heavily infested field near Amsterdam, MT, USA. Stubs containing larvae were subjected to storage temperatures of 0-4 C for 3-6 months to ensure completion of diapause before transfer to ventilated plastic Tupperware boxes (70 x 35 x 20 cm) at approximately 20 C for 4-5 weeks to facilitate adult emergence. Adult sawflies used in this study were less than 48 hours old and were not prevented from mating prior to experimentation.

Plants were infested as soon as the first internode was detected to ensure elongating stem tissue was present for oviposition, at approximately Zadoks growth stage 32 (Zadoks et al., 1974). This coincides with the earliest growth stage that sawfly females are likely to encounter and infest under field conditions. For each cultivar, three pots were randomly chosen for infestation and two pots were chosen for controls. Methods for infestation were adopted from Biyiklioglu et al., 2018. Briefly, a cage with 530 µM mesh openings was placed over the entire plant, including the main stem and any developing tillers and secured using a stake and wire. A 50/50 soil mix was added to the base of the chamber to prevent WSS escape. Supplementary lighting was placed approximately 45 cm on either side of the chamber to ensure that the sawflies were active (**Supplementary Figure 1**). Three WSS adult females were introduced to each chamber and oviposition was allowed for 72 hours, after which the chambers and sawflies were removed. Plant tissue was collected two weeks after the introduction of WSS to allow sufficient time for early instar larvae to cause damage to the lower internodes and maximize the likelihood that stem tissue in the upper internodes were free of frass. Specifically, the tissue was collected from upper internodes before more than one node could be bored by a small larva, so the upper internodes collected contained no frass. Sample tissue from upper internodes was not dissected in order to minimize damage to target plant tissues. To process samples, the main stem of the plant was cut at ground level and only the top two internodes were separated from the plant using a razor blade to ensure that stem tissue collected from infested plants was free of frass. The leaves were separated from the internodes, and internodes were wrapped in aluminum foil and immediately flash frozen in liquid nitrogen. Stems were considered infested if frass or larvae were observed in the lower internodes. Tillers were also checked for infestation, although most tillers had not reached the stem elongation stage during the infestation period. Three infested and three control samples were collected from individual plants for each cultivar and each sample was processed in 60 seconds or less. Samples were then stored in -80°C until metabolites were extracted.

### 2.2 Sample processing for metabolomics

Frozen wheat and oat stems were ground in liquid nitrogen with a mortar and pestle. Stem powder (approximately 150 mg per sample) was immersed in 100% methanol (MeOH) at 70°C for 15 min. Samples were vortexed for 1 min and then centrifuged (25,000 g, 10 min, 4°C). Proteins were separated from the metabolites by an acetone precipitation (two and a half parts acetone to one part tissue solution) at -80°C overnight, followed by centrifugation (25,000 g) at 4°C for 10 min. The resulting supernatant fraction was dried in a speed vacuum (low heat setting) and stored at -80°C. Prior to analyses by liquid chromatography-mass spectrometry (LC-MS), samples were resuspended in 20 µL of 50% HPLC grade water/50% MeOH.

### 2.3 LC-MS

Metabolite analysis was conducted at the Montana State Mass Spectrometry Facility using an Agilent 1290 ultra-performance liquid chromatography (UPLC) interface (Agilent Technologies, Santa Clara, CA, USA) fitted to an Agilent 6538 Accurate-Mass quadrupole time-of-flight mass spectrometer. Metabolites were separated by reversed-phase (RP) chromatography on a Kinetex 1.7 µm C18 150 x 2.1 mm column (Phenomenex, Torrance, CA) kept at 50°C with a flow rate of 600 µL min -1. The elution profile implemented started with a two-minute step of 98% solvent A (0.1% formic acid in H2O; waste) with 2% solvent B (0.1% formic acid in acetonitrile) followed by a 2% to 95% solvent B gradient over 24 min, a continued 95% solvent B for two min, and then a return to 2% solvent B over two min.

Mass detection was performed in positive mode, with a cone voltage of 3,500 V and a fragmentor voltage of 120 V. Drying gas temperature was 350°C (flow of 12 L per min -1) and the nebulizer was set at ~5.2 bar. Data was acquired with the following parameters: mass-to-charge ratio (m/z) range of 50-1,000 at 25,200 m/z-s. Mass analyzer resolution was 18,000 and post calibration tests had mass accuracy of approximately one ppm. For MS/MS acquisition, both standard compounds and ions of interest within a tolerance window of 1.7 m/z units were fragmented at 10 and 20 V.

### 2.4 LC-MS data preprocessing and analysis

Raw LC-MS data was converted to mzXML files using MSconvert from Proteowizard (Chambers et al., 2012). MZmine2 was used for preprocessing of spectral data (Pluskal et al., 2010). Raw data was acquired in centroid mode and was assigned m/z and retention time values using mass detection with noise level set at 1.0 E 3. Chromatograms were built using the chromatogram builder setting with minimum time span for peaks set to 0.1 min, minimum peak height set to 1.0 E 3 and m/z tolerance between 0.01 and 30 ppm. Resulting peaks were normalized using the same m/z tolerance, a retention time of 0.25 min and a minimum standard intensity of 2.0 E 4. Normalized peaks were then aligned, and the gap-filling feature was used to fill in missing peaks. Tentative identification of metabolites was completed using the online database metacyc.org (Katajamaa and Orešič, 2007; Pluskal et al., 2010; Caspi et al., 2014). Putatively identified metabolites were classified using the Chemical Entities of Biological Interest (ChEBI) database (http://www.ebi.ac.uk/chebi) (Hastings et al., 2016).

### 2.5 Statistical analysis

Data pre-processing and analysis was performed in R version 3.5.1 (Team, R. C 2022) using the R package MetaboAnalystR (Pang et al., 2020). Identified features were log transformed and range scaled to meet the assumption of normality. Normalized values were used to perform a principal component analysis (PCA) to identify which features contributed most to the variability of the dataset. To explore the response to infestation by each species as well as the effects of stem solidness, three related datasets were used: a complete dataset of all oat and wheat samples, an oat dataset containing infested and control samples from Dane and Otana, and a wheat dataset containing infested and control samples from Choteau, Scholar, Conan and Reeder. These three datasets were used to perform PCA as well as two-way ANOVA. For the complete dataset, two-way ANOVA was performed with infestation status (infested, control) and cultivar (Dane, Otana, Choteau, Scholar, Conan and Reeder) as fixed effects. Two-way ANOVA was also performed using the oat dataset with infestation status (infested, control) and cultivar (Dane, Otana) as fixed effects. Finally, the wheat dataset was used to perform a two-way ANOVA with infestation status (infested, control) and cultivar (Choteau, Scholar, Conan, Reeder) as fixed effects. For all ANOVA testing, *post-hoc* pairwise testing was performed using Fishers Least Significant Difference (LSD) test and false discovery rate (FDR) correction of p-values was used to correct for multiple comparisons.

## 3 Results

### 3.1 Complete dataset – oat and wheat

**Figure 1:**
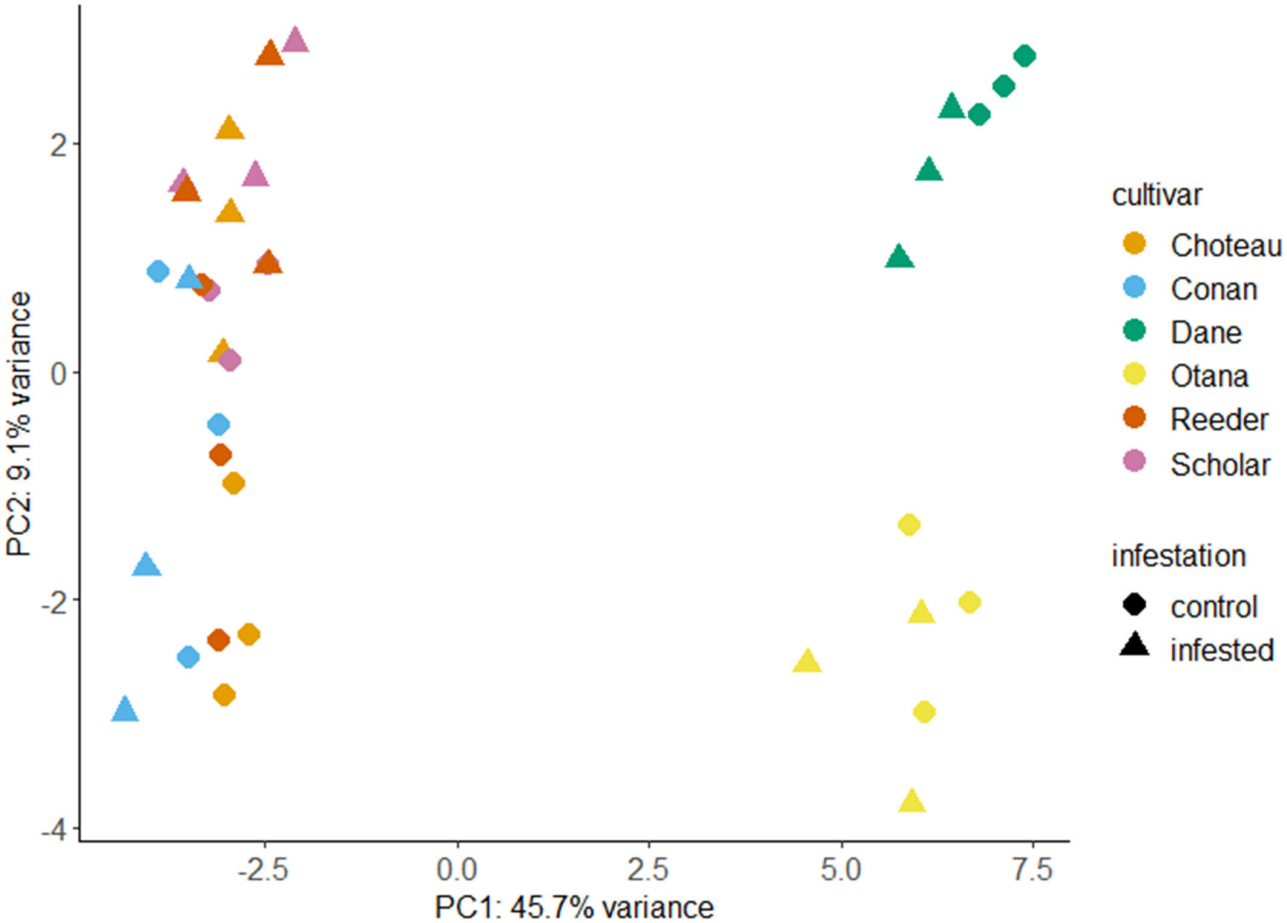
Principal component analysis (PCA) score plots for infested and control samples of all oat and wheat cultivars. PCA plots were created using LC-MS data from control and infested plants two weeks after caging with each point representing a stem sample. Choteau, orange; Conan, blue; Dane, green; Otana, yellow; Reeder, red; Scholar, Pink; Control, circle; Infested, triangle.

Prior to transformation of data, the complete dataset was used to calculate log2 fold changes between infested and control samples for each cultivar (**Supplementary Table 1**). After transformation, PCA utilizing all metabolites from the oat and wheat dataset showed clear separation of the oat and wheat samples based on the first principal component (PC1) which explained 45.7% of the variability in the dataset. The second principal component (PC2) explained 9.1% of the variability and was able to clearly discriminate between the oat cultivars. Some separation of the infested and control wheat samples was also observed based on PC2, with the exception of Conan samples which showed high variability in infested and control samples (**Figure 1**). The first principal component was defined by low values for several carbohydrates as well as some lipids and amino acids, and high values for several glycosides. The second principal component was defined by low values for a different set of carbohydrates, as well as some lipids and high values for some terpenoids. The first and second principal components were both defined by variable groups of compounds and not strongly defined by a particular class or classes of compound (**Supplementary Table 2**).

**Figure 2:**
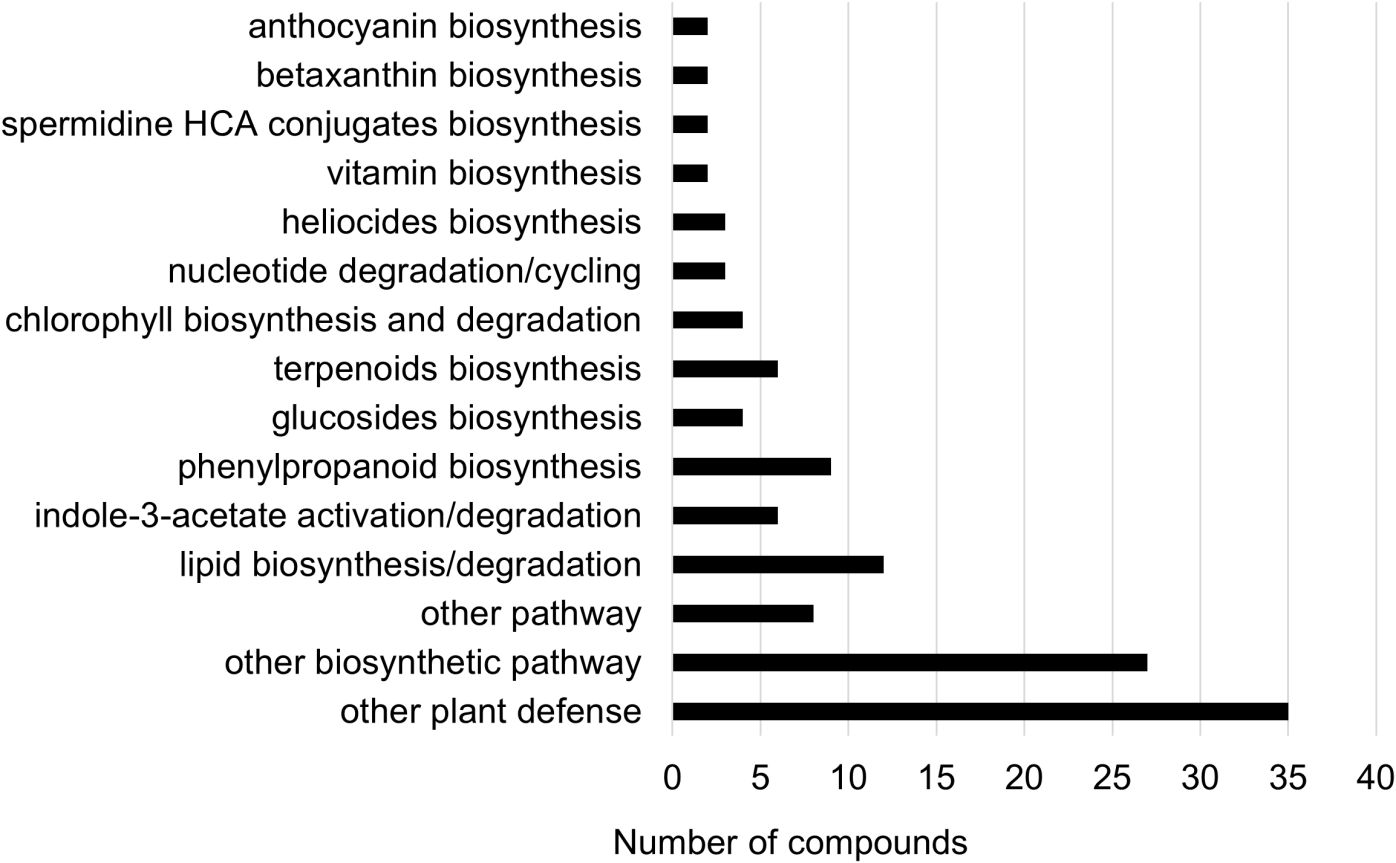
Biochemical pathway associations of significant compounds in the complete dataset which consisted of all oat and wheat samples from infested and control plants. Significant compounds (p-values<0.05) were identified using two-way ANOVA and Fishers LSD *post-hoc* test with FDR correction.

**Figure 3:**
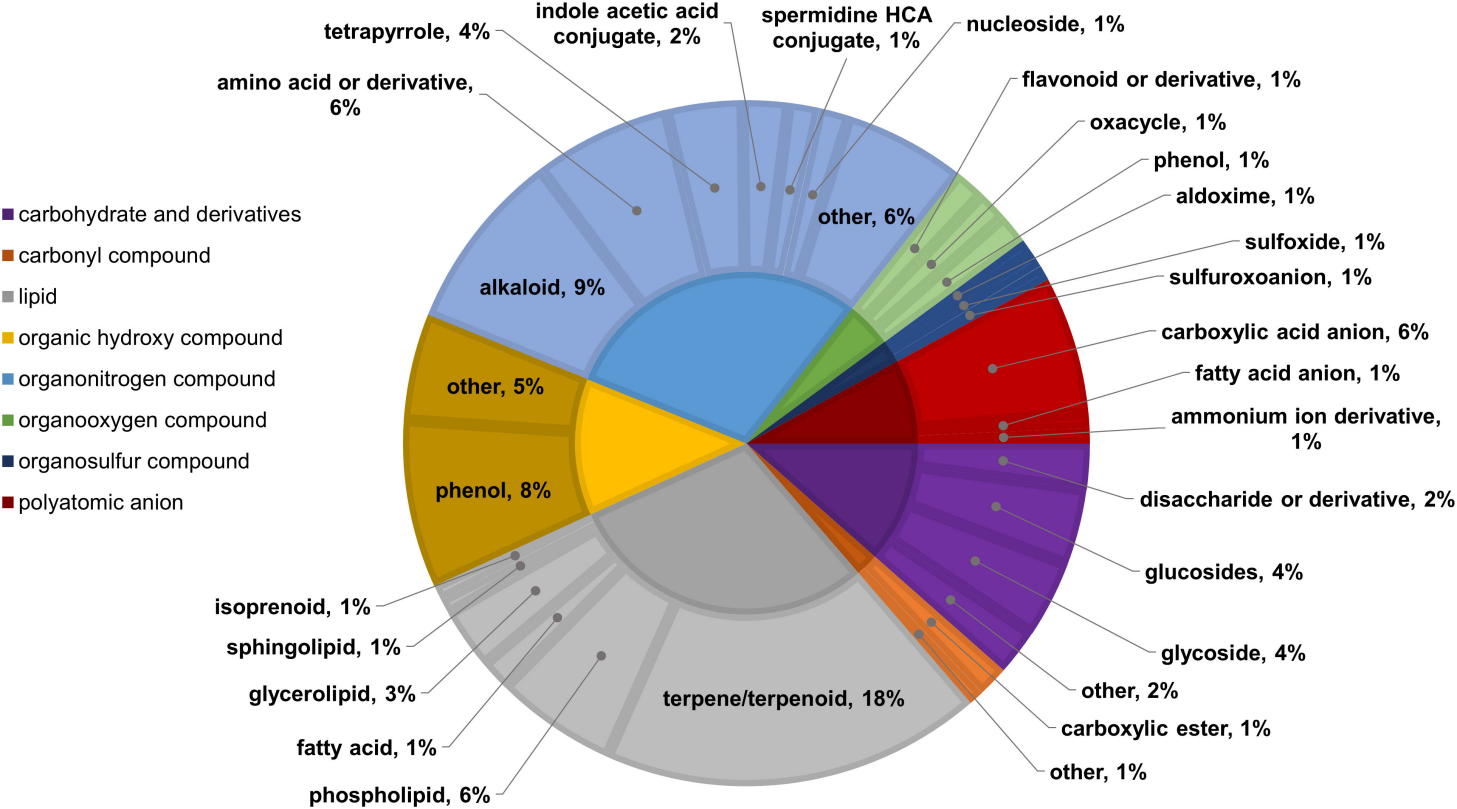
Classifications of significant putatively identified metabolites in the oat and wheat dataset. Significant compounds (p-values<0.05) were identified using two-way ANOVA and Fishers LSD *post-hoc* test with FDR correction. The innermost circle indicates the compound class with the outer ring representing compound subclass. Carbohydrate and derivatives, purple; carbonyl compound, orange; lipid, grey; organic hydroxy compound, yellow; organonitrogen compound, light blue; organooxygen compound, green; organosulfur compound, dark blue; polyatomic anion, red.

**Figure 4:**
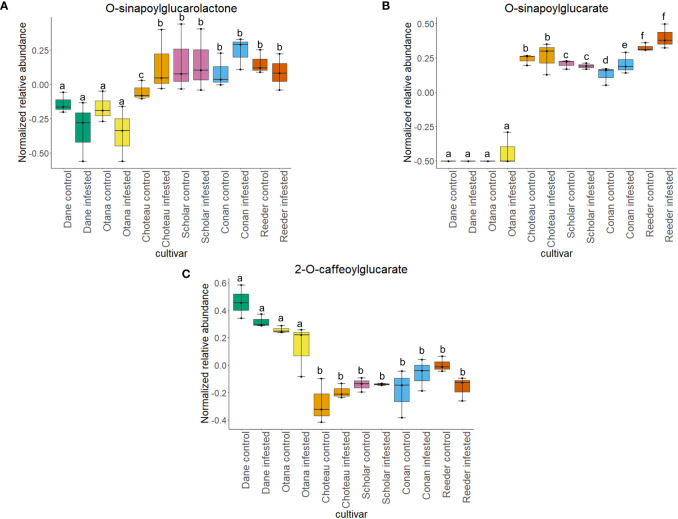
Boxplots showing differences in relative abundance of hydroxycinnamic acid conjugates in control and infested stem samples of oat and wheat. **(A)** O-sinapoylglucarolactone, **(B)** O-sinapoylglucarate, **(C)** 2-O-caffeoylglucarate. The box indicates the interquartile range (IQR) with the horizontal bar indicating the median. Lines extending from the box show the standard error for each sample group. Dane, green; Otana, yellow; Choteau, orange; Scholar, pink; Conan, blue; Reeder, red. Boxplots that do not share a letter have significantly different means.

**Figure 5:**
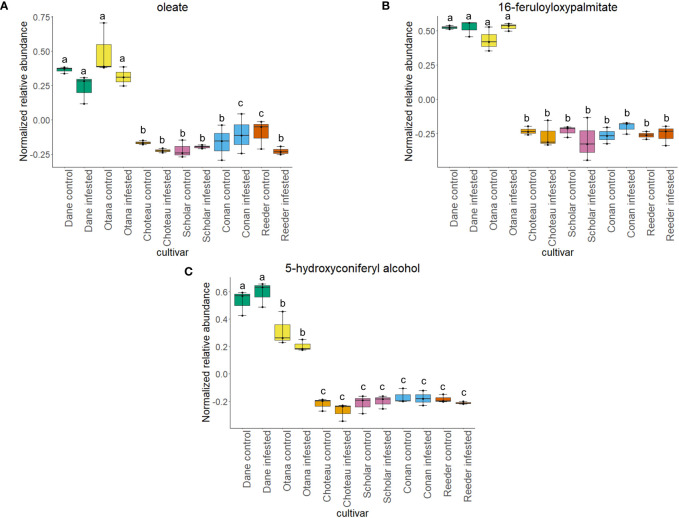
Boxplots showing relative abundance of compounds related to suberin and lignin biosynthesis in control and infested stem samples of oat and wheat. **(A)** oleate, **(B)** 16-feruloyloxypalmitate, **(C)** 5-hydroxyconiferyl alcohol. The box indicates the interquartile range (IQR) with the horizontal bar indicating the median. Lines extending from the box show the standard error for each sample group. Dane, green; Otana, yellow; Choteau, orange; Scholar, pink; Conan, blue; Reeder, red. Boxplots that do not share a letter have significantly different means.

**Figure 6:**
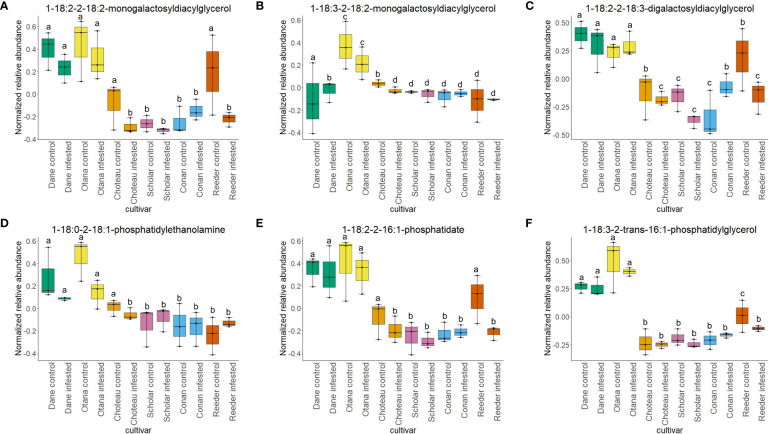
Boxplots showing relative abundance of phospho- and galactolipids in control and infested stem samples of oat and wheat. **(A)** 1-18:2-2-18-2-monogalactosyldiacylglycerol, **(B)** 1-18:3-2-18:2-monogalactosyldiacylglycerol, **(C)** 1-18:2-2-18:3-digalactosyldiacylglycerol, **(D)** 1-18:0-2-18:1-phosphatidylethanolamine, **(E)** 1-18:2-2-16:1-phosphatidate, **(F)** 1-18:3-2-trans-16:1-phosphatidylglycerol. The box indicates the interquartile range (IQR) with the horizontal bar indicating the median. Lines extending from the box show the standard error for each sample group. Dane, green; Otana, yellow; Choteau, orange; Scholar, pink; Conan, blue; Reeder, red. Boxplots that do not share a letter have significantly different means.

**Figure 7:**
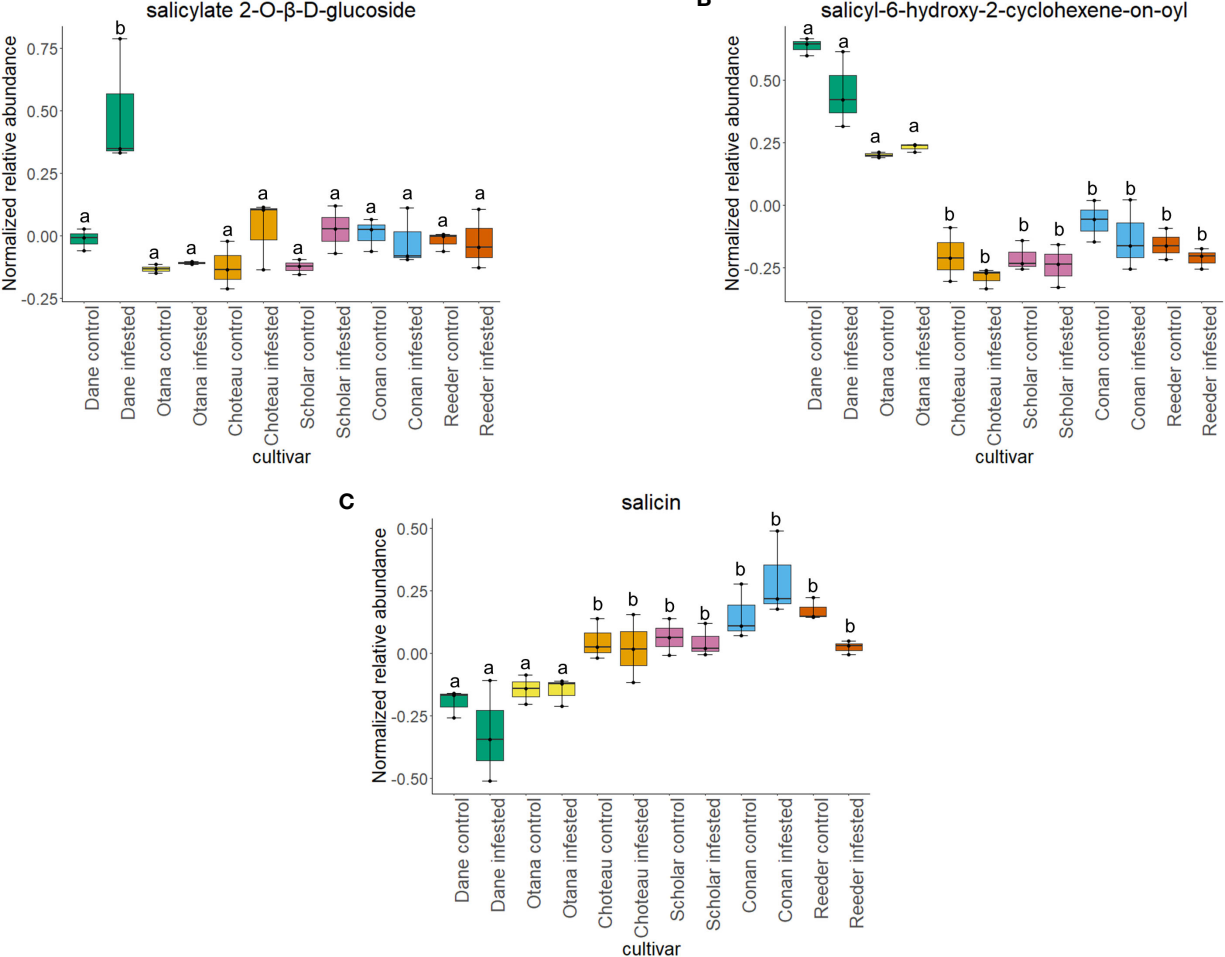
Boxplots showing relative abundance of salicylic acid related compounds in control and infested stem samples of oat and wheat. **(A)** salicylate 2-O-β-D-glucoside, **(B)** salicyl-6-hydroxy-2-cyclohexene-on-oyl, **(C)** salicin. The box indicates the interquartile range (IQR) with the horizontal bar indicating the median. Lines extending from the box show the standard error for each sample group. Dane, green; Otana, yellow; Choteau, orange; Scholar, pink; Conan, blue; Reeder, red. Boxplots that do not share a letter have significantly different means.

**Figure 8:**
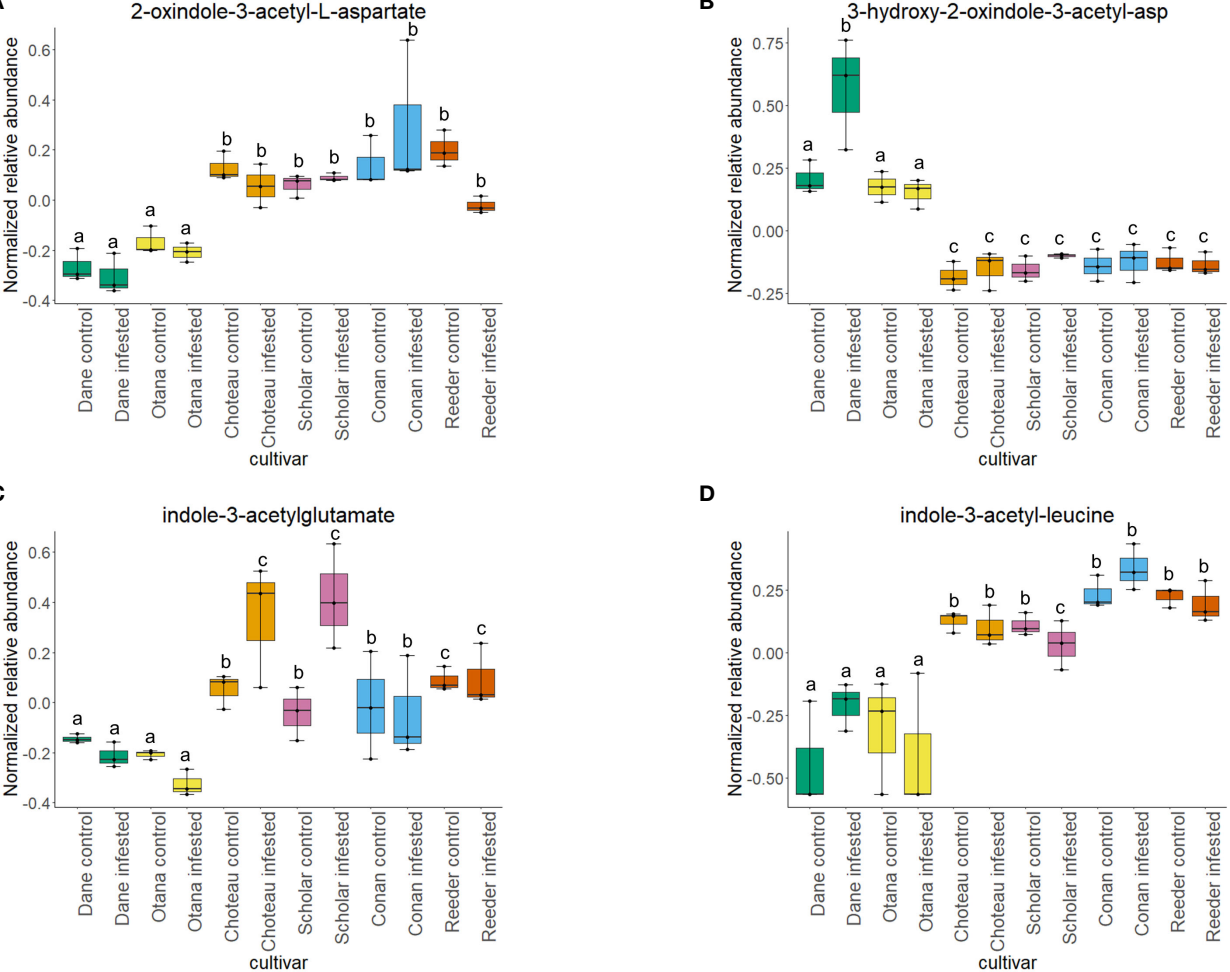
Boxplots showing relative abundance of indole-3-acetic acid (IAA) conjugates in control and infested stem samples of oat and wheat. **(A)** 2-oxindole-3-acetyl-L-aspartate, **(B)** 3-hydroxy-2-oxindole-3-acetyl-asp, **(C)** indole-3-acetylglutamate, **(D)** indole-3-acetyl-leucine. The box indicates the interquartile range (IQR) with the horizontal bar indicating the median. Lines extending from the box show the standard error for each sample group. Dane, green; Otana, yellow; Choteau, orange; Scholar, pink; Conan, blue; Reeder, red. Boxplots that do not share a letter have significantly different means.

**Figure 9:**
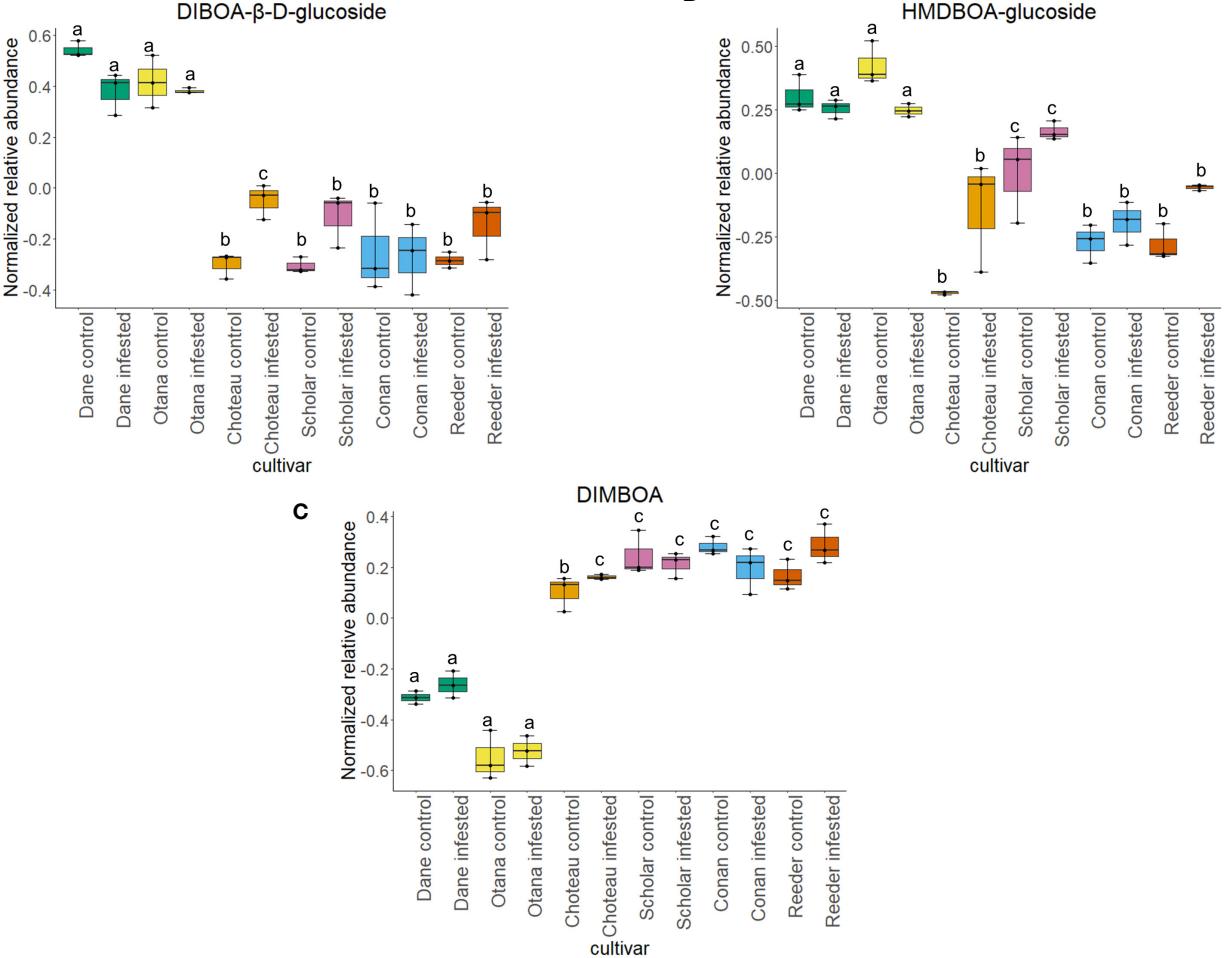
Boxplots showing relative abundance of benzoxazinoids in control and infested stem samples of oat and wheat. **(A)** DIBOA-β-D-glucoside, **(B)** HMDBOA-glucoside, **(C)** DIMBOA. The box indicates the interquartile range (IQR) with the horizontal bar indicating the median. Lines extending from the box show the standard error for each sample group. Dane, green; Otana, yellow; Choteau, orange; Scholar, pink; Conan, blue; Reeder, red. Boxplots that do not share a letter have significantly different means.

Two-way ANOVA of all oat and wheat samples identified 159 metabolites that showed significant differences between oat and wheat cultivars (p-value<0.05) (**Supplementary Table 3**). Of these compounds, it was possible to classify 141 and match 126 to their respective biological pathways. The majority of compounds were found to be directly involved in plant defense, including several related to glucosinolate biosynthesis and degradation (**Figure 2**). Groups of terpenoids, phenols, alkaloids, glycerolipids and phospholipids were also significantly higher in oat samples compared to wheat in both infested and control groups (p-values<0.05) (**Figure 3**). Fishers Least Significant Difference (LSD) *post-hoc* pairwise testing identified significant differences in abundance of several groups of related compounds in oat and wheat which were part of the same biological pathway (**Supplementary Table 4**). Compounds of interest were also chosen based on those identified in previous ‘omics studies of the WSS system and other studies of plant-insect interactions. Compounds O-sinapoylglucarolactone, O-sinapoylglucarate and 2-O-caffeoylglucarate, associated with the biosynthesis of hydroxycinnamate (HCA) esters were differentially expressed between oat and wheat cultivars, with O-sinapoylgluarate also showing significant differences among the wheat cultivars (**Figure 4**). Significantly higher abundance of oleate, 16-feruloyloxypalmitate and 5-hydroxyconiferyl alcohol, compounds associated with lignin and suberin biosynthesis, was observed in oat compared to all wheat cultivars, with significant differences in 5-hydroxyconiferyl alcohol also observed between oat cultivars Dane and Otana (**Figure 5**). Phospho- and galactolipids were also generally found in higher abundance in oat, including 1-18:2-2-18:2-monogalactosyldiacylglycerol, 1-18:3-2-18:2-monogalactodiacylglycerol, 1-18:2-2-18:3-digalactosyldiacylglycerol, 1-18:0-2-18:1-phosphatidylethanolamine, 1-18:2-2-16:1-phosphatidate and 1-18:3-2-trans-16:1-phosphatidylglycerol, however some infested and control samples of a few wheat cultivars were not significantly different from the oat cultivars for several of these lipids (**Figure 6**). Two-way ANOVA identified significant effects of cultivar and infestation status on the abundance of salicylic acid-related compound salicylate 2-O-beta-D-glucoside, as well as significant effects of cultivar on salicyl-6-hydroxy-2-cyclohexene-on-oyl and salicin (**Figure 7**). Compounds associated with indole acetic acid degradation and inactivation 2-oxindole-3-acetyl-L-aspartate, indole-3-acetylgluatamate and indole-3-acetyl-leucine were significantly lower in infested and control samples from oat cultivars compared to wheat, while 3-hydroxy-2-oxindole-3-acetyl-asp was significantly higher in oat cultivars, particularly in infested Dane samples (**Figure 8**). Benzoxazinoid-related glucoside DIBOA-beta-D-glucoside was found to be significantly higher in both Dane and Otana as well as in infested Choteau samples compared to other infested and control wheat samples (**Figure 9**).

### 3.2 Oat dataset

PCA using the oat dataset did not show any separation based on infestation status. The first principal component (PC1) explained 34.8% of the variability in the dataset and also allowed for discrimination of cultivars, while the second principal component (PC2) explained 15.7% of the variability in the dataset (**Supplementary Figure 2**). The first principal component was defined by low values for some amino acids and alkaloids and high values for some carbohydrate-related compounds. The second principal component was defined by low values for several lipids and high values for a different set of lipids as well as several carboxylic acids. The first and second principal components both were defined by a variable mix of compound classes (**Supplementary Table 5**).

Two-way ANOVA of all oat samples identified 96 metabolites that were significantly different between cultivars (p-value<0.05) (**Supplementary Table 6**). Of these, it was possible to classify 81 and match 79 to their respective biological pathway. The majority of these metabolites were involved in plant defense or biosynthetic pathways including nucleotide cycling and degradation, phosphatidylcholine biosynthesis and biosynthesis of secondary metabolites (**Supplementary Figure 3**). Compounds that were significantly different between the oat cultivars included several alkaloids, phenols, terpenes, flavonoids and carbohydrates (**Supplementary Figure 4**).

### 3.3 Wheat dataset

PCA with only the wheat dataset showed Choteau, Conan, Reeder and Scholar samples forming distinct clusters, but overlap between groups was observed due to the variability within sample groups (**Supplementary Figure 5**). The first principal component (PC1) explained 19.7% of the variability in the dataset and while the second principal component (PC2) explained 16.3%. Overall, Scholar samples showed the tightest clustering, with lower variability observed in infested and control groups when compared with the other cultivars. The first principal component was defined by small values for some amino acids, lipids and polyatomic ions and large values for some carbohydrates and carbohydrate derivatives. The second principal component was defined by small values for some lipids, carbohydrates and organic hydroxy compounds and large values for a different set of lipids and some phenols. As with the complete dataset and oat dataset, neither principal component was strongly defined by any specific compound classes (**Supplementary Table 7**).

**Figure 10:**
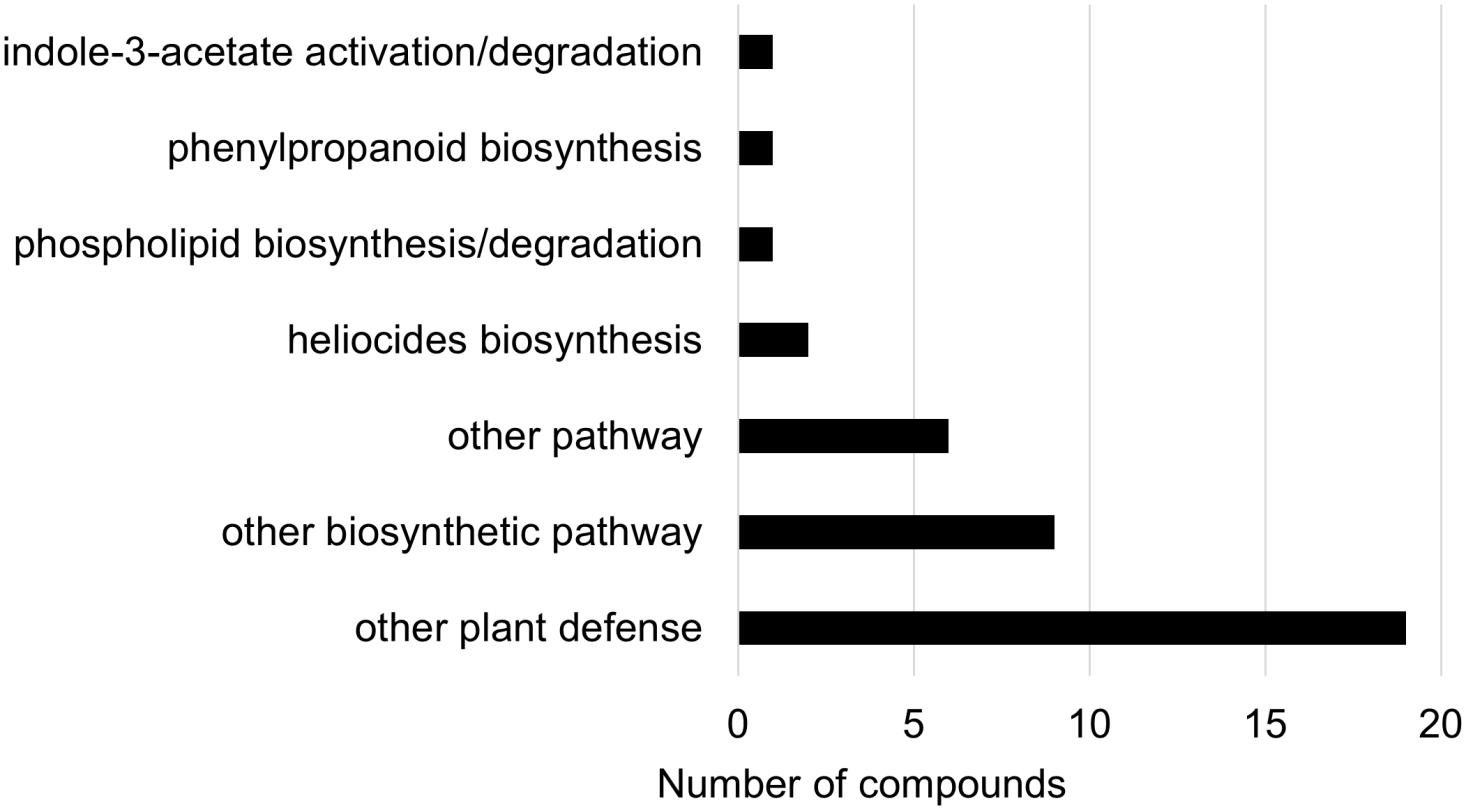
Biochemical pathway associations of significant compounds (p-value<0.05) in the wheat dataset. The wheat dataset consisted of all wheat samples from infested and control plants. Significant compounds (p-values<0.05) were identified using two-way ANOVA and Fishers LSD *post-hoc* test.

**Figure 11:**
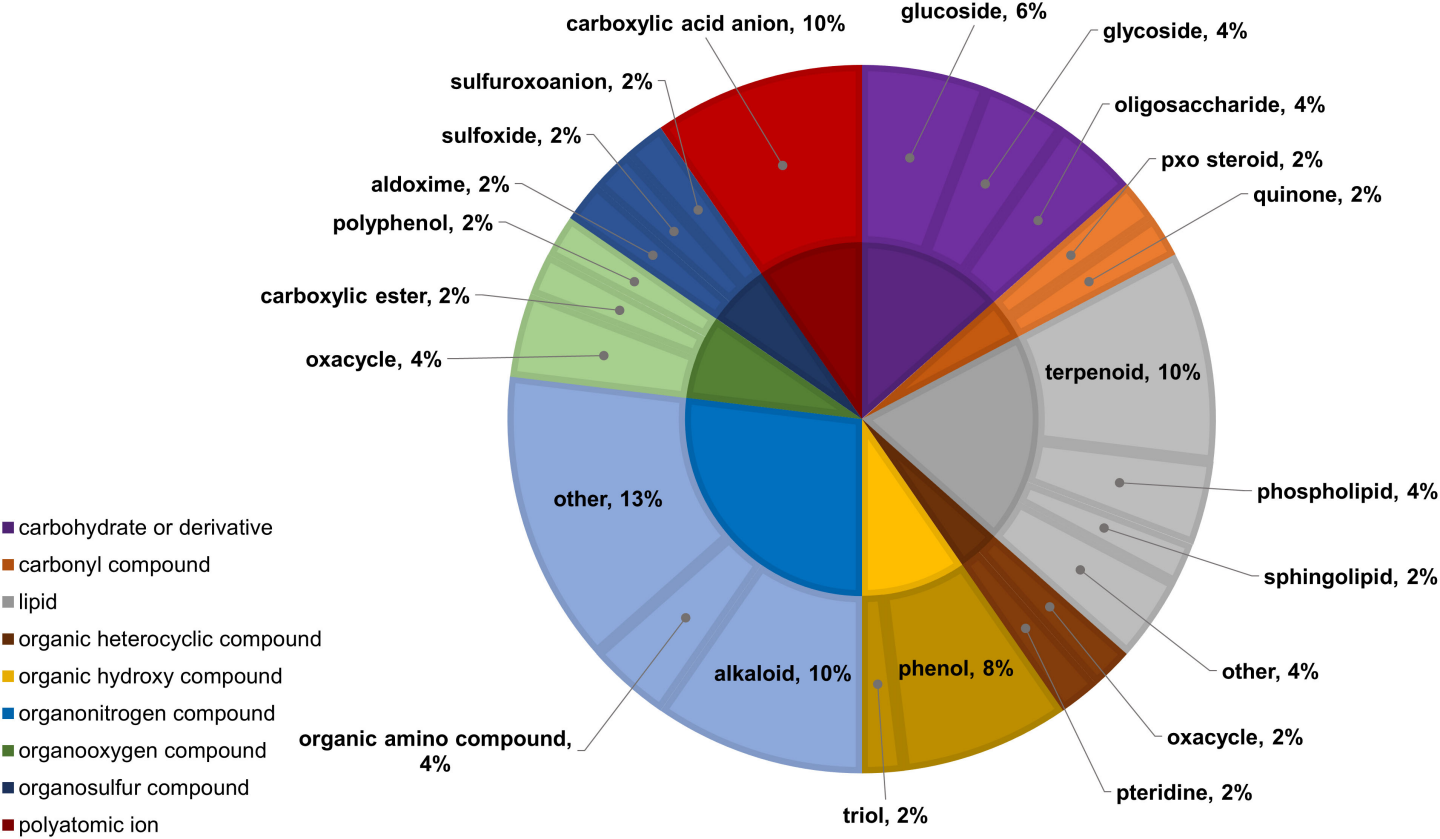
Classifications of significant, putatively identified metabolites in the wheat dataset. Significant compounds (p-values<0.05) were identified using two-way ANOVA and Fishers LSD *post-hoc* test with FDR correction. The innermost circle indicates the compound class with the outer ring representing compound subclass. Carbohydrate or derivative, purple; carbonyl compound, orange; lipid, grey; organic heterocyclic compound, brown; organic hydroxy compound, yellow; organonitrogen compound, light blue; organooxygen compound, light green; organosulfur compound, dark blue; polyatomic ion, red.

In the two-way ANOVA of wheat samples, a total of 58 metabolites showed significant differences. Cultivar had a significant effect on the abundance of 52 compounds and infestation status had a significant effect on 11 compounds, with evidence for an interaction between cultivar and infestation status for three compounds (p-value<0.05) (**Supplementary Table 8**). Of the 58 total compounds, it was possible to classify 52 and assign 39 to their respective pathway (**Figures 10**, **11**). Metabolites that were significantly different between cultivars included several carbohydrate and carbohydrate derivatives, lipids and organonitrogen compounds, while infestation status had a significant effect on some lipids and carbohydrate related compounds.

### 3.4 Carbohydrates and derivatives

In the complete dataset, 17 carbohydrates, carbohydrate derivatives and carbonyl compounds were differentially expressed between cultivars, including salicin 6-phosphate, salicin, DIBOA β-D-glucoside and 2-O-caffeoylglucarate (p-values <0.05). In *post-hoc* tests, salicin showed significant differences between all samples from oat cultivars compared to all cultivars of wheat (**Figure 7**). 2-O-caffeoylglucarate and DIBOA beta-D-glucoside were both significantly higher in samples from oat cultivars compared to wheat, with significantly higher abundance of DIBOA beta-D-glucoside also observed in infested samples of Choteau compared to samples from other wheat cultivars (**Figures 4**, **9**). There was also evidence for an interaction between cultivar and infestation status for four compounds: tetrahydropteroyltri-L-glutamate, vitexin 2”-O-β-D-glucoside, benzoyl-β-D-glucopyranose and phlorizin (p-values <0.05).

In the oat dataset, there was also a significant effect of cultivar on 11 carbohydrate-related compounds including salicortin, 2-O-caffeoylglucarate, peonidin-3-(p-coumaroyl)-rutinoside-5-glucoside and 1,4-β-D glucan (p-values <0.05). Infestation status and cultivar both had significant effects on the relative abundance of glucosyl limonin and benzoyl-β-D-glucopyranose in the oat dataset, with evidence of an interaction between infestation status and cultivar for these compounds (p-values <0.05).

The wheat dataset contained 9 glucosides and carbonyl compounds which were differentially expressed between cultivars (p-value <0.05). Infestation status had a significant effect on bis(β-D-glucosyl) crocetin (p-value <0.05). Both cultivar and infestation status had a significant effect on the abundance of HMDBOA-glucoside, but there was no evidence of an interaction for this compound (p-value <0.05). Abundance of HMDBOA-glucoside was higher in all samples from oat cultivars compared with infested and control samples from wheat. Scholar infested and control samples were significantly higher than those from other wheat cultivars (**Figure 9**).

### 3.5 Lipids

In the complete dataset, 40 lipids were differentially expressed between cultivars, including several galactolipids, phospholipids and terpenoids (p-values<0.05). 1-18:2-2-18:2-monogalactosyldiacylglycerol, 1-18:3-2-18:2-monogalactodiacylglycerol, 1-18:2-2-18:3-digalactosyldiacylglycerol, 1-18:0-2-18:1-phosphatidylethanolamine and 1-18:2-2-16:1-phosphatidate and 1-18:3-2-trans-16:1-phosphatidylglycerol showed differential expression (**Figure 6**). Oleate was significantly higher in the oat samples compared to wheat, and was also significantly higher in infested Conan samples and control samples of Reeder compared to other samples from wheat (**Figure 5**). Infestation status also had a significant effect on the abundance of 2-omega-hydroxy- C22:0-LPA and hemigossypol, although there was no evidence of an interaction between cultivar and infestation status for these compounds (p-value<0.05).

There were 10 lipids in the oat dataset that were differentially expressed between cultivars, including oleate, the galactolipids 1-18:3-2-18:2-monogalactosyldiacylglycerol and 1-18:2-2-18:2-digalactosyldiacylglycerol and several terpenoids (p-value<0.05).

In the wheat dataset, cultivar had a significant effect on the abundance of 9 lipids, including the phospholipid 1-18:3-2-trans-16:1-phosphatidylglycerol. Infestation status had a significant effect on the abundance of 2-omega-hydroxy- C22:0-LPA (p-value<0.05). There was no evidence for an interaction between cultivar and infestation status for any lipids in the wheat dataset.

### 3.6 Other organic compounds

Cultivar had a significant effect on the abundance of 100 other organic compounds in the complete dataset, including 16-feruloyloxypalmitate, salicyl-6-hydroxy-2-cyclohexene-on-oyl, 2-oxindole-3-acetyl-L-aspartate, 3-hydroxy-2-oxindole-3-acetyl-asp, phenols O-sinapoylglucarolactone and 5-hydroxyconiferyl alcohol, and amino acid derivatives oxindole-3-acetyl-L-aspartate, indole-3-acetyl-glutamate and indole-3-acetyl-leucine (p-value<0.05). Indole-3-acetylglutamate and indole-3-acetyl-leucine were both significantly lower in oat samples compared with all cultivars of wheat (**Figure 8**). Indole-3-acetylglutamate also showed differential expression between infested and control samples of Choteau and Scholar (**Figure 8**). 16-feruloyloxypalmitate and 5-hydroxyconiferyl alcohol were significantly higher in both oat cultivars compared with wheat (**Figure 5**). 2-oxindole-3-acetyl-L-aspartate and O-sinapoylglucarolactone were significantly lower in both Dane and Otana compared with the wheat cultivars (**Figures 8**, **4**). 3-hydroxy-2-oxindole-3-acetyl-asp showed opposite expression, with significantly higher abundance in Dane infested samples, although all oat samples showed higher abundance than the wheat cultivars (**Figure 8**). Infestation status had a significant effect on the abundance of 4 organic compounds, including indole-3-acetyl isoleucine and salicylate 2-O-β-D-glucoside (p-value<0.05). Salicyl-6-hydroxy-2-cyclohexene-on-oyl was significantly higher in both oat cultivars compared with the wheat cultivars, however there were also differences observed between oat cultivars for this compound (**Figure 7**). Salicylate 2-O-β-D-glucoside was significantly higher in Dane infested samples compared with other oat samples and wheat samples from all cultivars (**Figure 7**). There was evidence for an interaction between cultivar and infestation status for tetrahydropteroyl-tri-L-glutamate, 10-deacetyl-2-debenzoylbaccatin III, hydroxyflavone and kamepferide triglycoside (p-value<0.05).

In the oat dataset, 62 organic compounds were differentially expressed between cultivars including DIMBOA, choline, cyanidin 3-(p-coumaroyl)-glucoside, indole-3-acetyl-isoleucine (p-value <0.05). Infestation status had a significant effect on the abundance of 6-hydroxy-allocryptopine and indole-3-acetohydroximoyl-glutathione (p-value <0.05), however there was evidence of an interaction between infestation status and cultivar for these compounds (p-value<0.05).

In the wheat dataset, 36 organic compounds were differentially expressed between cultivars including O-sinapoylglucarate and several phenols and alkaloids (p-value<0.05). Infestation status had a significant effect on the abundance of 4 compounds: serotonin, xanthine, dihydrocurcumin and 7-deoxyloganetate (p-value<0.05). There was evidence for an interaction between infestation status and cultivar for two compounds: laudanosine and luteolin 7-O-β-D-glucuronide (p-value<0.05).

## 4 Discussion

Here, we utilized comparisons of the metabolome of infested and uninfested stem tissue from two cultivars of oat, which exhibits complete resistance to stem cutting by WSS, and four cultivars of wheat with varying degrees of stem solidness to explore possible mechanisms of resistance to WSS. In our complete dataset, several carbohydrates, lipids and plant defense compounds were differentially expressed between samples from both oat cultivars and samples from wheat cultivars, indicating that these compounds may be responsible for the constitutive resistance to WSS observed in oat. In all datasets, cultivar had the greatest effect on most compounds, and although some did show change in response to WSS infestation, the effect of infestation status on relative abundance was rarely significant. Overall, constitutive and induced differences in lipids, plant defense compounds including HMDBOA glucoside, and compounds involved in the organization of cells or cell elongation within plant tissue such as O-sinapoylglucarate and indole-3-acetyl leucine appear to be related to WSS resistance and illustrate how the molecular environment of oat stems differ from wheat and may explain why oat is a poor host for developing larvae of WSS.

### 4.1 Phenylpropanoid pathway and species differences in lignin and suberin biosynthetic pathways

The phenylpropanoid pathway is extensive and complex, producing a wide range of compounds which are involved in plant defense in several ways including systemic signaling and providing chemical or physical barriers to insect or pathogen attack. This pathway is responsible for the biosynthesis of hydroxycinnamic acids, flavonoids, isoflavanones, anthocyanins, and sinapate esters (Strack et al., 1987). Several structural biopolymers are derived from the phenylpropanoid pathway as well, including lignin and suberin. Production of these compounds is highly variable depending on species and nearly every class of molecule produced by this superpathway has functions that are either directly or indirectly involved in plant defense (Dixon et al., 2002).

Biosynthesis of hydroxycinnamate (HCA) esters proceeds via three unique routes: one utilizing HCA-CoA thioesters, another uses HCA-glucosides from sinapate ester biosynthesis and the third utilizes HCA-quinate (Strack et al., 1987; Strack et al., 1988). O-sinapoylglucarate and O-sinapoylglucarolactone, both products of the second route of HCA ester biosynthesis, were found in lower abundance in oat samples when compared to wheat samples. In wheat, abundance of O-sinapoylglucarate and O-sinapoylglucarolactone increased or stayed the same when plants were infested, while infested oat plants had a significant decrease in O-sinapoylglucarolactone compared to uninfested oat plants (**Figure 4**). Overall, 2-O-caffeoylglucarate, a product of the third route of HCA ester biosynthesis, had increased abundance in oat samples compared to wheat and also had significantly lower abundance in infested oat plants compared to uninfested oat plants (**Figure 4**). These results indicate that oat may produce HCA esters primarily via the HCA-quinate route. It is not known whether the routes to HCA production differ in terms of efficiency or speed of response, so it is difficult to determine what role the HCA-quinate route plays in oat resistance to larval feeding.

Hydroxycinnamic acid esters in plant tissues have a direct effect on plant resistance and can decrease oviposition and have a negative effect on larval mortality (Anyanga et al., 2021). They can also form polymers with polyamines to form hydroxycinnamic acid amines (HCAAs) which are deposited in the cell wall near regions of pathogen infection or wounding by boring insects, a process that is associated with strengthening of cell walls and increased resistance (Grandmaison et al., 1993; Facchini et al., 2002; Barros-Rios et al., 2011; Gunnaiah et al., 2012). HCAAs themselves can influence cell growth and lignin content as well as lignin subunit composition, which also affects plant resistance (Lima et al., 2013).

Suberin and lignin are structural polymers that are essential for plant protection from infection by fungal pathogens or infestation by insects (Wainhouse et al., 1990; Li et al., 2007; Kosma et al., 2010; Bakaze et al., 2020). Infection by some pathogens induces reinforcement of vascular cell walls with a ligno-suberin coating that strengthens the cell wall, preventing degradation and restricting the movement of the pathogen, conferring resistance (Kashyap et al., 2022). In wheat, lignin is present in the parenchyma of solid-stemmed cultivars, while lignification is not as extensive in barley or oat (Roemhild, 1954). In our complete dataset, abundance of 16-feruloyloxypalmitate, oleate, and 5-hydroxyconiferyl alcohol were significantly lower in wheat compared to all oat cultivars (**Figure 5**). 16-feruloyloxypalmitate and oleate are predicted to be involved in suberin biosynthesis, while 5-hydroxyconiferyl alcohol is incorporated into lignin via a dehydrogenation reaction, forming lignins that are linear in nature (Elder et al., 2016). While several studies have confirmed the positive impact of increased lignin on plant resistance to fungal pathogens and infestation by insects (Wainhouse et al., 1990; Bi et al., 2010), lignin content has not been found to have any effect on the amount of sawfly tunneling or stem cutting in the sawfly system (McNeal et al., 1965).

However, total lignin content is only one aspect of mechanical resistance to pests and pathogens. Lignin is a polymer composed of syringyl (S), guaiacyl (G) and p-hydroxyphenyl (H) subunits which may have an effect on resistance. In particular, some research has explored the influence of lignin subunit composition (S/G ratio) on palatability of plant tissue to insect herbivores, and there is evidence that resistant plants exhibit an altered S/G ratio depending on species (Sewalt et al., 1997; Poerschmann et al., 2005; Kärkönen et al., 2014). Lignin formed in response to bacterial infection or insect attack contains less G monolignin and more G and H monolignins, illustrating that biosynthesis of lignin can change depending on type of stress, though this does not always coincide with increased plant resistance (Zhang et al., 2007; Buhl et al., 2017). Further study is necessary to elucidate whether the lignin composition of oat differs from that of wheat and whether this has an effect on larval feeding or survival in oat.

### 4.2 Phospholipids in oat and wheat

Phospholipids such as phosphatidylcholine and phosphatidylethanalomine are important structural lipids found in plant cell membranes. These molecules can also be hydrolyzed by phospholipases and serve as co-factors, signaling molecules or precursors to signaling molecules that are necessary for plant defense responses (Munnik et al., 1998; Laxalt and Munnik, 2002). Phospholipids, galactolipids and related molecules found in this study have the potential for involvement in plant defense responses in some capacity and may be linked to the same putative pathway (**Figure 6**). Several phospholipid metabolizing enzymes have been identified and are thought to play an important role in plant defense signal transduction. A known product of phospholipases C (PLC; EC 3.1.4.3) and D (PLD; EC 3.1.4.4) is phosphatidic acid which is a conjugate acid of phosphatidate (Liscovitch et al., 2000). In many plants, levels of phosphatidic acid have been shown to increase in response to wounding, drought stress or treatment with a general synthetic elicitor (Lee et al., 1997; Frank et al., 2000; Van Der Luit et al., 2000). Phosphatidic acid is also a precursor to many complex lipids including digalactosyldiacylglycerol, which in turn contributes to salicylic acid biosynthesis and systemic acquired resistance (SAR) signal azelaic acid (Gao et al., 2014). Phosphatidic acid can also activate the NADPH oxidase complex, indirectly triggering an oxidative burst in response to pathogen attack (Laxalt and Munnik, 2002).

In this study, both species experienced a decrease in phospholipid abundance in response to infestation, with the exception of Conan, which showed an increase in concentration for the majority of phospholipids observed. Lipid components of plant cellular membranes, 1-18:1-2-18:1-phosphatidylethanolamine, 1-18:0-2-18:1-phosphatidylethanolamine and 1-18:3-2-trans-16:1-phosphatidylglycerol, were significantly greater in oat compared with all cultivars of wheat. In the oat dataset, free galactolipids 1-18:2-2-18:2-digalactosyldiacylglycerol and 1-18:3-2-18:2-monogalactosyldiacylglycerol both decreased in response to infestation, but only cultivar had a significant effect on abundance. Lipids, including phospholipids, are nutritionally significant to many insects and are often required for normal growth and development (Turunen, 1979). Conversely, high concentrations of lipids in plant tissues can also cause larval mortality and decreased larval growth in some species (Li et al., 2014; Liao et al., 2021). WSS larvae are adapted to feeding inside the plant stem, making it difficult to test their dietary requirements with artificial diets, so little is known about their specific nutritional needs, but it is possible that higher levels of lipids in oat may have a negative effect on larval growth and development (Macedo et al., 2005).

Phospholipids have been well studied in relation to wheat resistance to Hessian fly, an insect that feeds within the exterior of the stem wall. In several studies using wheat lines resistant to Hessian fly, a decrease in membrane lipid concentration and increase in fatty acids and derivatives has often been observed in response to feeding by Hessian fly larvae (Zhu et al., 2012; Khajuria et al., 2013). In our results, infestation did not have a significant effect on the levels of phospho- or galactolipids in any of the datasets, although a decrease of abundance was observed in infested samples, indicating that phospholipid signaling is likely not a strong response to WSS infestation in either oat or wheat (**Figure 6**). The resistant cultivar Conan had similar levels of structural lipids to the other wheat cultivars but had increased abundance of free lipids 1-18:2-2-18:2-monogalactosyldiacylglycerol, 1-18:2-2-18:2-digalactosyldiacylglycerol, 1-18:2-2-18:3-digalactosyldiacylglycerol and 1-18:1-2-18:1-phosphatidate, though this increase was not significant in either the complete or wheat-only dataset (**Figure 6**). These results suggest that the increase in free lipids might be associated with the disappearance of pith observed as Conan matures and indicate that Conan may be more efficient than other wheat cultivars at re-mobilizing structural lipids after degradation to signaling lipids or precursors. The downstream effects of increased phosphatidic acid, including SAR and induction of an oxidative burst, may also partially explain the resistance of Conan to sawfly damage.

### 4.3 Other plant defense molecules

#### 4.3.1 Salicylic acid and related metabolites

Salicylic acid (SA) and jasmonic acid (JA) hormone signaling pathways are the two most important and well-studied in relation to induced plant defense (Howe and Jander, 2008; Erb et al., 2012). The crosstalk between these pathways is complex, but SA can have a negative effect on JA-induced resistance, making plants more susceptible to damage from insect herbivores (Stotz et al., 2002; Bodenhausen and Reymond, 2007; Bruessow et al., 2010). Salicylates such as salicin and salicortin also play a role in direct plant defense against herbivory, negatively affecting larval performance by decreasing growth rates (Lindroth and Peterson, 1988; Lindroth and Bloomer, 1991; Hemming and Lindroth, 2000; Zhang et al., 2013). Additionally, there is evidence that activation of jasmonate and salicylic acid defenses can increase parasitism or predation of herbivores in many plant species (Dicke et al., 1990; Thaler, 1999; Van Poecke and Dicke, 2002).

In this study, there was a strong effect of cultivar on the abundance of the salicylic acid-related compounds salicylate 2-O-β-D-glucoside, salicin and salicyl-6-hydroxy-2-cyclohexene-on-oyl, while infestation status only had an effect on the levels of salicylate 2-O-β-D-glucoside in infested Dane samples. There was no significant effect of cultivar or infestation status on the abundance of salicortin, however, salicyl-6-hydroxy-2-cyclohexene-on-oyl, the final precursor in salicortin biosynthesis was significantly higher in both oat cultivars (**Figure 7**). Salicortin itself did decrease in all cultivars of oat and wheat in response to infestation, but this response was not found to be significant. Regardless, as salicyl-6-hydroxy-2-cyclohexene-on-oyl is likely more stable than salicortin *in vivo*, the increased abundance of salicyl-6-hydroxy-2-cyclohexene-on-oyl may indicate an upregulation of the salicortin biosynthesis pathway in oat. In a study of willow *Salix sericea* (Marsh.) subjected to damage by herbivory from various species of beetle, salicortin decreased in the affected leaves four days after the start of damage, possibly due to consumption of leaf tissue high in this compound (Fields and Orians, 2006). Since the tissue collected for our study was very likely to be undamaged by larval feeding, but was collected after larvae were able to feed within the stem for some time, the decrease in salicortin is more likely due to a systemic response or degradation as opposed to a short term induced defense response. Salicortin may be remobilized to damaged regions of the stem where it is rapidly degraded into salicin during larval feeding, which might explain the decrease in levels of salicortin in systemic stem tissue of infested samples.

In the complete dataset, cultivar had an effect on abundance of only one compound derived from the jasmonates biosynthesis pathway, 12-hydroxyjasmonic acid 12-O-β-D-glucoside (tuberonic acid glucoside, TAG), while infestation status had no measurable effect. Two additional jasmonate-related compounds, jasmonoyl-CoA and jasmonoyl-L-isoleucine, were identified in the complete dataset, but cultivar and infestation status had no effect on abundance (data not shown). While not a significant result, TAG decreased with infestation in Choteau and Conan with no change in infested Scholar or Reeder. Conversely, both cultivars of oat showed an increase in TAG abundance in infested samples, although this result was not significant in the complete or oat dataset. After JA is converted to TAG in damaged regions of the plant, it is translocated to undamaged plant tissue where it contributes to down-regulation of genes involved in JA biosynthesis, which may explain the insignificant effect of WSS infestation on JA-associated compounds identified in our analysis (Gidda et al., 2003; Seto et al., 2009).

#### 4.3.2 DIMBOA pathway

2,4-dihydroxy-7-methoxy-1,4-benzoxazin-3-one (DIMBOA) and related 1,4-benzoxazin-3-ones (Bxs) are related to the resistance of cereal species to insects, fungi and bacteria (Niemeyer, 1988). These metabolites are present in plant cells as glucosides, which become available to hydrolyzing enzymes once plant tissues are damaged by insect feeding (Virtanen and Hietala, 1960). Bxs have recently been discovered to play a key role in the stem elongation phase in winter wheat (Xu et al., 2021). Much of the research conducted on this class of molecules has focused on increased levels of DIMBOA-Glc, 2-(2-hydroxy-7-methoxy-1,4-benzoxazin-3-one)-β-D-glucopyranose (HDMBOA-Glc) and 6-methoxy-2-benzoxazolinone (MBOA) in maize resistance to European corn borer, *Ostrinia nubilalis*, however Bxs have also been found to be involved in plant resistance to several species of aphids, as well as larvae from many species (Campos et al., 1989; Niemeyer et al., 1989; Houseman et al., 1992; Ortego et al., 1998). Evidence from previous untargeted proteomics and metabolomics experiments show that Bxs may also be involved in plant response to WSS in spring and winter wheat (Biyiklioglu et al., 2018; Lavergne et al., 2020).

A metabolite putatively identified as DIMBOA was included in our analysis, but abundance was only affected by cultivar in the oat dataset with no differences in the wheat or complete datasets. While all wheat cultivars experienced an increase in abundance of HDMBOA-Glc after infestation, the increase in Conan was insignificant compared to Choteau, Scholar and Reeder (**Figure 9**). HDMBOA-Glc can be induced during feeding by *O. nubilalis* or upon treatment with jasmonic acid (JA) or chitin derived saccharides in wheat and maize (Oikawa et al., 2001; Oikawa et al., 2002; Dafoe et al., 2011). After hydrolysis of HDMBOA-Glc, HDMBOA decomposes rapidly into MBOA at a faster rate than other known Bxs, and both HDMBOA and MBOA have negative effects on larval growth of Southwestern corn borer, *Diatraea grandiosella* Dyar (Grambow and Lückge, 1986; Hedin et al., 1993). This means that HDMBOA-Glc may be involved in the rapid defense response of wheat to WSS, although it appears that Conan may return to baseline levels more quickly than other cultivars.

In our complete dataset, DIBOA-β-D-glucoside had significantly higher abundance in both oat cultivars compared to all wheat cultivars. While not significant, wheat cultivars experienced an increase in DIBOA-β-D-glucoside upon infestation while both oat cultivars showed a decrease of this metabolite after infestation (**Figure 1**). The increased abundance of DIBOA-β-D-glucoside in infested wheat samples observed here matches the plant response observed in the hollow-stemmed partially tolerant cultivar Hatcher reported in figures of a previous study of plant metabolomic response to WSS (Lavergne et al., 2020). Conversely, we observed a decrease in DIBOA-β-D-glucoside upon infestation in all oat cultivars. In a separate study of the proteome of infested spring wheat cultivars, downregulation of DIMBOA glucoside β-D-glucosidase, a protein which hydrolyzes DIMBOA-glucoside to DIMBOA and D-glucose, was identified in infested stems of Scholar, but no change was observed in resistant cultivar Choteau (Biyiklioglu et al., 2018). These conflicting results illustrate the complex involvement of this pathway in WSS resistance and are not unexpected, since Bxs vary significantly based on cultivar, growth stage, plant part sampled and time after infestation (Niemeyer et al., 1989; Huang et al., 2006). Adding to the complexity of this response, there is some evidence that Bxs in the diet of cereal aphid species can have a positive effect on aphid predators, maximizing the tritrophic interaction between wheat, aphids and aphid predator *Eriopis connexa* Germar. (Martos et al., 1992). This means that Bxs in the diet of WSS larvae have the potential to impact the fitness of specialist braconid parasitoids that have WSS as their only known host. This possibility, along with the association of Bxs in plant response to WSS infestation and with the specific response of the resistant cultivar Conan show that further exploration of this pathway will be beneficial to understanding WSS resistance.

#### 4.3.3 Indole-3-acetate degradation

Phytohormone indole-3-acetic acid (IAA), is the primary auxin found in plants, and is involved in many aspects of plant development and response including tropism, cell elongation and stem growth, cell division, phloem and xylem differentiation, root initiation, flowering, senescence and abscission (Davies, 2010). Concentrations of IAA in plant tissues is regulated by the plant in several ways, including biosynthesis, degradation, activation, transport in and out of cells, and inactivation via formation of IAA conjugates (Caruso, 1987; Bartel et al., 2001). Despite knowledge of the existence of these regulatory mechanisms, the exact pathways for many are still only partially elucidated (Ljung, 2013). IAA can also be produced by some species of insects in high concentrations, allowing them to form galls, increase nutritive value of plant tissue or overcome structural plant defenses (Tooker and De Moraes, 2010; Tooker and De Moraes, 2011; Dafoe et al., 2013).

In the complete dataset, cultivar had a strong effect on the abundance of several IAA-conjugates and IAA-derived compounds that are involved in indole-3-acetate degradation, while infestation status had no significant effect on the abundance of these compounds. Abundance of 2-oxindole-3-acetyl-L-aspartate, indole-3-acetylglutamate, and indole-3-acetyl-leucine had significantly lower abundance in oat samples compared to wheat, while 3-hydroxy-2-oxindole-3-acetyl-aspartate was significantly higher in oat samples (**Figure 8**). The function of IAA-leucine appears to be temporary storage to supply free IAA when needed by the cell (for IAA-activation), so the lower abundance observed in oat may indicate that free IAA can be readily provided by degradation of IAA-leucine or IAA-glutamate in wheat (Woodward and Bartel, 2005). IAA-glutamate is also involved in an indole-3-acetate conjugate degradation pathway and may serve as a temporary storage form of IAA since it is easily reconverted to IAA *in vivo* (Hayashi et al., 2021). IAA-glutamate can also be irreversibly oxidized to 2-oxindole-3-acetyl-L-aspartate and further hydrolyzed to 2-oxindole-3-acetic acid, an inactive form of IAA (Hayashi et al., 2021). 3-Hydroxy-2-oxindole-3-acetyl-aspartate was found in lower abundance in oat, with the exception of Dane infested samples. This compound may be involved in degradation of 2-oxindole-3-acetic acid aspartate to 3-O-β-glucosyl-2-oxindole-3-acetyl-aspartate, but it is unknown whether this mechanism can be reversed to provide free IAA (Caspi et al., 2014).

The significantly lower abundance of IAA-glutamate and the decrease that was observed in infested oat, while not significant, suggests that oat may not inactivate IAA to the same extent as in wheat, instead storing it in forms that can be re-activated. In fact, with the exception of Conan, all wheat cultivars experienced an increase in IAA-glutamate upon infestation and also had higher abundance of 2-oxindole-3-acetyl-L-aspartate, the product of the irreversible step of IAA inactivation in both infested and uninfested tissue samples. These results indicate that wheat may respond to WSS infestation by inactivating IAA in the stem, while fully irreversible inactivation of IAA is more variable depending on cultivar.

Free IAA, whether produced by the plant or by insect, can affect insect survival not only through physical consumption, but by altering plant host material for protection or increased nutrient availability. Hessian fly infestation causes elevated levels of IAA without a corresponding induction of jasmonic acid and salicylic acid, leading to increased nutrient availability while simultaneously lowering plant defense activation (Tooker and De Moraes, 2010). In maize, high levels of IAA are excreted in the frass of European corn borer, leading to increased protein accumulation in nearby tissues which provide larvae with more nutritional content, offsetting the decline in growth rates caused by the presence of increased plant defense compounds (Dafoe et al., 2013). IAA application to the larvae of lesser wax moth, *Achoria grisella* not only has an effect on lipid, protein and glycogen levels in the hemolymph of the pest, but also affects hemolymph content of its braconid natural enemy *Apanteles galleriae* (Uçkan et al., 2014). These changes can even have a negative effect on natural enemy populations, increasing adult emergence time after pupation and decreasing overall longevity of *A. galleriae* adults (Uçkan et al., 2011). These studies indicate that further exploration of IAA biosynthesis and degradation pathways may provide more information about the tritrophic interaction between wheat, WSS and their parasitoids.

## 5 Conclusion

The solid stem phenotype has historically been the most utilized defense against damage and yield loss from WSS infestation, but due to variability in solid stem expression under different growing conditions, and the negative effect of solid stems on braconid parasitoid populations, substantial stem cutting and yield loss can still occur. To support parasitoid populations and maximize the effectiveness of the tritrophic interaction between host, pest and parasitoid, it is necessary to explore new sources of resistance which are not associated with solid stems. Oat exhibits complete resistance to WSS, causing 100% larval mortality to early instar larvae, which makes it a prime candidate for investigating new mechanisms of host plant resistance.

Here, we identified differentially expressed metabolites between cultivars of oat and wheat which were associated with changes to the phenylpropanoid pathway and phospholipid biosynthesis and signaling. Compounds such as O-sinapoylglucarate, O-sinapoylglucarolactone, 2-O-caffeoylglucarate (all are HCAs) and 5-hydroxyconiferyl alcohol are ubiquitous in plants and are generally related to cellular organization and tissue structure. Due to their presence in stem tissue, HCAs as well as membrane and free lipids are directly encountered by foraging females during ovipositor insertions as well as by larvae feeding inside the stem. HCAs can also affect oviposition behavior and larval survival by strengthening the cell wall and inducing changes in overall lignin content and composition, while lipids can serve as signaling molecules, affecting a wide range of downstream defense responses. Additionally, increased abundance of 5-hydroxyconiferyl alcohol in oat suggests that there may be differences in lignin subunit composition that is related to WSS resistance. In oat, several phospho- and galacto- lipids were also found in greater abundance, and both species experienced a decrease in phospholipid abundance after infestation, with the exception of Conan, which has the early stem-solidness phenotype and loses pith as the plant develops. Other studies have also identified a role for the phenylpropanoid pathway in plant defense against wheat stem sawfly, and changes in lipid abundance have known associations with plant resistance in other systems, making these pathways good targets for further exploration (Zhu et al., 2012; Khajuria et al., 2013; Biyiklioglu et al., 2018; Lavergne et al., 2020). Further examination of lignin content and composition in hollow, solid, and early-solid cultivars may reveal differences in stem architecture that influence the behavior of both adult and larval WSS. Additionally, more reliable methods for observing larval behavior in laboratory conditions will also allow for rigorous testing of the dietary requirements of WSS larvae to determine how lipid content or lignin subunit composition might affect larval growth and development.

Differentially expressed metabolites in the response of oat and wheat to infestation by WSS were also found which were associated with the salicylic acid signaling pathway, IAA degradation, and biosynthesis of 1,4-benzoxazin-3-ones (Bxs). Some salicylic acid related compounds were found in higher abundance in oat samples of both cultivars, indicating that salicylic acid pathway defenses may be involved in the defense response to WSS in oat. Additional compounds related to plant defense that were identified in this study indicate the potential for defining the relationships between wheat, WSS and their parasitoids *B. cephi* and *B. lissogaster*. Bxs HDMBD-Glc and DIBOA-β-D-glucoside increased in all wheat cultivars after infestation with the exception of Conan, with oat expressing overall higher abundance of both compounds. Wheat also appears to respond to WSS infestation by inactivating IAA in stem tissues, while irreversible inactivation of IAA is dependent on cultivar. Further study of the effect of 1,4-benzoxazin-3-ones on sawfly development is necessary, especially since other studies of the sawfly system have identified changes in DIMBOA and related compounds in plants infested with WSS and the results have been highly variable (Biyiklioglu et al., 2018; Lavergne et al., 2020). There is evidence that both 1,4-benzoxazin-3-ones and IAA pathway-related compounds not only have an effect on larval pests but can either positively or negatively affect parasitoid populations so it is unclear what this means for braconid parasitoid populations in the WSS tritrophic system.

Ultimately, though solid stems do offer resistance to the plant, continued exploration of the tritrophic interaction between wheat, WSS and their parasitoids will help in mitigating negative effects on this system to maximize the success of control efforts. Additionally, increased abundance of metabolites in oat which are related to lignin biosynthesis and composition as well as increased abundance of several phospho- and galactolipids illustrate the need for further investigation of stem architecture at the molecular level as well as examination of the developmental and behavioral effects these metabolites have on WSS at all stages of development.

## Data availability statement

The original contributions presented in the study are included in the article/**Supplementary Material**. Further inquiries can be directed to the corresponding author.

## Author contributions

MSH: Formal analysis, Writing – original draft. MLH: Data curation, Investigation, Writing – review & editing. AV: Data curation, Investigation, Writing – review & editing. BB: Data curation, Investigation, Writing – review & editing. HB: Conceptualization, Writing – review & editing. DW: Conceptualization, Supervision, Writing – review & editing.

## Supplementary material

Supplementary Figure 1 Experimental setup for greenhouse infestation by WSS

Supplementary Figure 2 Principal component analysis (PCA) score plots for oat cultivars Dane and Otana. PCA plots were created using LC-MS data from control and infested plants two weeks after caging with each point representing a stem sample. Dane, green; Otana, yellow; Control, circle; Infested, triangle.

Supplementary Figure 3 Oat dataset - Biochemical pathway associations of significant compounds in the oat dataset. The oat dataset consisted of all oat samples from infested and control plants. Significant compounds (p-values<0.05) were identified using two-way ANOVA and Fishers LSD *post-hoc* test.

Supplementary Figure 4 Classifications of significant, putatively identified metabolites in the oat dataset. Significant compounds (p-values<0.05) were identified using two-way ANOVA and Fishers LSD *post-hoc* test with FDR correction. The innermost circle indicates the compound class with the outer ring representing compound subclass. Carbohydrate and derivatives, purple; carbonyl compound, orange; lipid, grey; organic heterocyclic compound, brown; organic hydroxy compound, yellow; organonitrogen compound, light blue; organooxygen compound, light green; organophosphorus compound, dark green; organosulfur compound, dark blue; polyatomic ion, red.

Supplementary Figure 5 Principal component analysis (PCA) score plots for wheat cultivars Choteau, Conan, Reeder and Scholar. PCA plots were created using LC-MS data from control and infested plants two weeks after caging with each point representing a stem sample. Choteau, orange; Conan, blue; Reeder, red; Scholar, pink; Control, circle; Infested, triangle.

Supplementary Table 1 Log2 fold changes between infested and control samples of oat and wheat.

Supplementary Table 2 PCA loadings for the oat and wheat complete dataset.

Supplementary Table 3 Significant compounds identified from the oat and wheat complete dataset. Significant compounds (p-values<0.05) were identified using two-way ANOVA and Fishers LSD *post-hoc* test with FDR correction.

Supplementary Table 4 Results from Fisher’s LSD *post-hoc* test.

Supplementary Table 5 PCA loadings for the oat dataset.

Supplementary Table 6 Significant compounds identified from the oat dataset. Significant compounds (p-values<0.05) were identified using two-way ANOVA and Fishers LSD *post-hoc* test with FDR correction.

Supplementary Table 7 PCA loadings for the wheat dataset.

Supplementary Table 8 Significant compounds identified from the wheat dataset. Significant compounds (p-values<0.05) were identified using two-way ANOVA and Fishers LSD *post-hoc* test with FDR correction.

Supplementary Table 9 Complete oat and wheat dataset. Relative abundances of each compound for all oat and wheat stem samples, m/z ratio, retention time and predicted formula.
